# Evaluation of La(XT), a novel lanthanide compound, in an OVX rat model of osteoporosis

**DOI:** 10.1016/j.bonr.2021.100753

**Published:** 2021-02-11

**Authors:** Yunyun Di, Ellen K. Wasan, Jacqueline Cawthray, Jaweria Syeda, Munawar Ali, David M.L. Cooper, Ahmad Al-Dissi, Nima Ashjaee, Wubin Cheng, James Johnston, David M. Weekes, Thomas I. Kostelnik, Chris Orvig, Kishor M. Wasan

**Affiliations:** a College of Pharmacy and Nutrition, University of Saskatchewan, 104 Clinic Place, Saskatoon, SK S7N 2Z4, Canada; b Department of Anatomy Physiology and Pharmacology, College of Medicine, University of Saskatchewan, 107 Wiggins Road, Saskatoon, SK S7N 5E5, Canada; c Western College of Veterinary Medicine, University of Saskatchewan, 52 Campus Drive, Saskatoon, SK S7N 5B4, Canada; d College of Engineering, University of Saskatchewan, 57 Campus Drive, Saskatoon, SK S7N 5A9, Canada; e Medicinal Inorganic Chemistry Group, Department of Chemistry, University of British Columbia, 2036 Main Mall, Vancouver, BC V6T 1Z1, Canada; f Department of Urologic Sciences, Faculty of Medicine, University of British Columbia, Vancouver, British Columbia, Canada

**Keywords:** OVX, ovariectomized, Ca^2+^, calcium, La^3+^, lanthanum, Cr, creatinine, CRF, chronic renal failure, ALT, alanine aminotransferase, AST, aspartate aminotransferase, BMD, bone mineral density, SD, Sprague Dawley, HAP, hydroxyapatite, BV/TV, bone volume fraction, Tb.N, trabecular number, Tb.Sp, trabecular separation, Tb.Th, trabecular thickness, Osteoporosis, Lanthanum, La(XT), OVX, Toxicity

## Abstract

**Purpose:**

The purpose of this study was to evaluate the efficacy and toxicity of a novel lanthanum compound, La(XT), in an ovariectomized (OVX) rat model of osteoporosis.

**Methods:**

Twenty-four ovariectomized female Sprague Dawley rats were divided into 3 groups receiving a research diet with/without treatment compounds (alendronate: 3 mg/kg; La(XT) 100 mg/kg) for three months. At the time of sacrifice, the kidney, liver, brain, lung and spleen were collected for histological examination. The trabecular bone structure of the tibiae was evaluated using micro-CT and a three-point metaphyseal mechanical test was used to evaluate bone failure load and stiffness.

**Results:**

No significant differences were noted in plasma levels of calcium, phosphorus, creatinine, alanine aminotransferase (ALT), and aspartate aminotransferase (AST) between the La(XT) treatment compared to the non-treated OVX group. Alendronate-treated animals (positive control) showed higher BV/TV, Tb.N and lower Tb.Th and Tb.Sp when compared to the non-treated OVX group. Mechanical analysis indicated that stiffness was higher in the alendronate (32.88%, p = 0.04) when compared to the non-treated OVX group. Failure load did not differ among the groups.

**Conclusions:**

No kidney or liver toxicities of La(XT) treatments were found during the three-month study. The absence of liver and kidney toxicity with drug treatment for 3 months, as well as the increased trabecular bone stiffness are encouraging for the pursuit of further studies with La(XT) for a longer duration of time.

## 1 Background

Osteoporosis is a systemic bone disorder characterized by microarchitectural deterioration of bone tissue and loss of bone density (Diab and Watts, 2013). According to the International Osteoporosis Foundation report, at least one in three women and one in five men over age 50 will experience an osteoporotic fracture during their lifetime all over the world (Foundation IO, n.d.). To date, the pharmacological treatment of osteoporosis that utilizes multiple medications including bisphosphonates, hormone therapy, selective estrogen receptor modulators (SERMs), monoclonal antibodies (denosumab) and parathyroid hormones, each of which has a place in therapy by promoting osteoblast activity or as anti-resorptive agents via inhibiting the osteoclast reabsorption of bone mass. Likewise, each has unique limitations, risks or toxicities such that new anti-osteoporosis drugs are still needed (Cawthray et al., 2017; Tu et al., 2018; Cheng et al., 2020; Li et al., 2020; Cosman et al., 2016).

Compounds of metal ions have been receiving attention as bone-seeking agents because they deposit into hydroxyapatite by exchange of calcium (Ca^2+^) through biological processes, thus leading to the accumulation of metal ions in bone tissue (Pemmer et al., 2013). The effect of metal ions on bone microstructure depends on their natural properties. Ions of metals such as lead, mercury, chromium, cobalt and cadmium are associated with bone microstructure impairment and a lowering of bone mineral density (Lim et al., 2016; Scimeca et al., 2017). Nonetheless, alkaline earth metal ions such as barium and strontium exhibit high binding affinities to hydroxyapatite (HAP), resulting in an increased bone mineral density and an improvement in bone strength. It has been demonstrated that both barium and strontium target to bone where active bone formation and bone remodeling are taking place; this has been elucidated using 3D synchrotron K-edge subtraction micro-CT imaging (Panahifar et al., 2018). Barium is highly toxic to the cardiovascular system due to potassium channel blockade, thus it is highly unlikely to be developed as a therapeutic bone-seeking agent (Bhoelan et al., 2014). Strontium promotes osteoblast activity and bone formation, with 99% of absorbed strontium depositing into bone tissue following parental administration (Dahl et al., 2001; Blaschko et al., 2013; Hamdy, 2009; Quade et al., 2020). In clinical trials, strontium ranelate showed a moderate anti-osteoporotic effect and a reduced risk of vertebral and non-vertebral fractures in postmenopausal women with osteoporosis (Reginster et al., 2005; Meunier et al., 2002). Strontium ranelate (Protelos®) was marketed in Europe for the treatment of osteoporosis in 2004, but its use was restricted after ten years due to an elevated risk of cardiovascular events and venous thromboembolism (Sundar et al., 2014). Therefore, there is still room in the therapeutic arsenal for an alternative and less toxic bone-seeking metal ion compound with anti-osteoporotic activity.

The lanthanides are a series of trace metal elements that also well-suited for ion exchange with Ca^2+^, with a high affinity to HAP (Cawthray et al., 2015). Evidence in vitro, has shown that lanthanum itself (La^3+^) promotes osteogenesis, activating osteoblast differentiation, while inhibiting the differentiation and activation of osteoclast cells (Wang et al., 2008; Harini et al., 2014; Zhang et al., 2018; Jiang et al., 2016). Animal studies found that La^3+^ accumulated in the liver, femur and kidney with supplementation of lanthanum carbonate [La_2_(CO_3_)_3_] after 22 weeks, with an average of 1322 ng/g wet weight of La^3+^ detected in the femoral head in a rat model of chronic renal failure (CRF) (Damment et al., 2011). The concentration of La^3+^ in bone was maximized to 2 μg/g wet weight in the rats treated with 2000 mg/kg/day of orally administered La_2_(CO_3_)_3_ for 8 weeks (Bervoets et al., 2006). Upon deposition into bone, La_2_(CO_3_)_3_ is localized at the outer edge of mineralized bone along the trabeculae as well as deep inside trabecular bone and deposited in the mineral component of the matrix (Behets et al., 2005; Yu et al., 2017). There are multiple effects of La^3+^ on bone. It has been suggested that La^3+^ may cause a reversible mineralization defect in CRF rats, without affecting active cuboidal osteoblasts in bones (Bervoets et al., 2006). The implications for human bone, however, are not clear at this time. Meanwhile, little effect of La^3+^ on bone mineral density was observed in healthy animals (Damment et al., 2011). In another study that utilized ovariectomized rats as an osteoporotic model, it was reported that a three-month dietary supplementation of La^3+^ significantly increased tibial trabecular bone density in the animals treated with either La_2_(CO_3_)_3_ or lanthanum citrate. The serum level of osteocalcin, a bone formation marker, was also significantly elevated in these animals (von Rosenberg and Wehr, 2012).

In human medicine, a La^3+^ containing compound, La_2_(CO_3_)_3_, is used as phosphorus binder to treat hyperphosphatemia in late stage renal failure patients. La_2_(CO_3_)_3_ is therefore well known to be safe and well-tolerated during long-term oral administration even though accumulation of La^3+^ was observed in liver, kidney and bone (Hutchison et al., 2008; Toussaint et al., 2020; Zhang et al., 2013). In an open-labelled comparator-controlled phase III clinical trial, the study reported no elevated liver enzyme activities in hemodialysis patients receiving 375–3000 mg/day La_2_(CO_3_)_3_ for two years (Hutchison et al., 2006). Plasma La^3+^ concentration reached plateau between 0.4 and 0.6 ng/mL and notably declined 6 weeks after discontinuation of the treatment (Hutchison et al., 2006; Spasovski et al., 2006). Bone concentration of La^3+^, also significantly increased after 1-year treatment with La_2_(CO_3_)_3_, showing a mean of 2.3 ± 1.6 μg/g (Spasovski et al., 2006). A kinetic model predicted that La^3+^ deposited into trabecular bone at a yearly rate of 1.03 ± 0.23 μg/g wet bone and had a fractional loss of 13.3 ± 5.0% per year after cessation (Bronner et al., 2008). A Japanese study showed that bone La^3+^ level increased to 4.3 μg/g after three years which was comparable with the valued predicted from the kinetic model (Shigematsu et al., 2011). The effects of La^3+^ in bones have not been fully elucidated, but clinical studies suggest a beneficial effect. To our knowledge, there are no clinical trials published which are specifically designed to measure the effect of La_2_(CO_3_)_3_ on bone structure. However, it is notable that a 2-year open label study reported that La_2_(CO_3_)_3_ improved bone formation rate (74.2% vs 57.1%) and bone volume fraction (84.4% vs 62.5%) in patients with end stage renal disease, compared to a calcium carbonate group (Malluche et al., 2008). La_2_(CO_3_)_3_ has also been shown to improve the bone turnover rate after 18 months of treatment in hemodialysis patients, which found that serum osteocalcin levels were significantly lower compared with patients treated with calcium carbonate (Goto et al., 2019).

The major drawback of La^3+^ usage clinically is that the aqueous solubility of lanthanum is extremely low. Lanthanum is only soluble in an acidic environment while the pH within gastrointestinal tract (GIT) is alkaline where the vast majority of absorption takes place. La3+ within the GIT binds to phosphate to form insoluble lanthanum phosphate resulting in a lower GI absorption. The bioavailability of lanthanum was estimated to be 0.00127% in human and 0.0007% within rats, confirming that La^3+^ was very poorly absorbed (Damment and Pennick, 2008; Pennick et al., 2006; Yang et al., 2013). We believed that the low oral bioavailability of lanthanum may be due to the low solubility of the lanthanum compound. Thus, our laboratories have developed several lanthanum complexes, which have enhanced aqueous solubility. Two of our lead compounds, La(ddp)_3_ and La(XT) showed excellent binding to hydroxyapatite without altering its lattice structure (Barta et al., 2007; Mawani et al., 2013). Biodistribution studies in female Sprague Dawley (SD) rats revealed that both La(ddp)_3_ and La(XT) accumulated in bone with La(XT) displaying a greater bone accumulation comparing with La(ddp)_3_ (Cawthray et al., 2015; Weekes et al., 2017). Moreover, an in vitro study found that La(XT) affected bone remodeling by decreasing the tartrate-resistant acid phosphatase activity and inhibiting the differentiation of RAW 264.7 into osteoclast-like cells at 2.5–100 μM/L (data not published). A preliminary in vivo study was performed in our laboratory in which La(dpp)_3_ and La(XT) were administered orally to rats for 30 days. No significant changes in plasma aspartate aminotransferase (AST), alanine aminotransferase (ALT) nor creatinine were observed, which suggested a lack of significant liver or kidney toxicity at La(XT) doses of up to 200 mg/kg/day for 4 weeks (Weekes et al., 2017). La(dpp)_3_ was deemed a less suitable candidate due to poor solubility and low bioavailability, therefore further studies were only pursued with La(XT). These preliminary results suggested that La(XT) may be safely administered with preferable accumulation into bone. However, it was necessary to evaluate the toxicity profile of La(XT) in an osteoporosis model with a longer duration of administration. Thus, we conducted a three-month study in an ovariectomized osteoporosis rat model (OVX) with daily oral administration of La(XT). This study presented here also evaluates the effect of La(XT) on plasma biomarkers of bone turnover as well as comparative bone microstructure analysis.

## 2 Materials

K_2_La(XT)·3.75H_2_O with 21.85% La^3+^ was synthesized accord to previously published literature (Cawthray et al., 2015). Alendronate sodium trihydrate, 10% neutral buffered formalin, 100% ethanol, 24 gauge catheter, 1 mL Luer-Lok™ syringes, 4 mL BD vacutainer blood collection tubes, 0.9% saline, and DMSO were purchased from Thermo Fisher Scientific Canada (Saskatoon, SK).

### 2.1 Methodologies

#### 2.1.1 Animal study

Twenty-four female SD rats (226–385 g body weight, and 4 months old) (Charles River, Montreal, Canada) were housed as pairs in the University of Saskatchewan laboratory animal services unit with a 12-h light and 12 h-darkness cycle. All animals had free access to a basic rat chow diet (Research Diets Inc., Montreal Quebec, Canada), with/without the test compound La(XT) (described below), as well as *ab libitum* water throughout the study period. After one week of acclimation, OVX rats (n = 24) were randomized into 3 groups: i) non-treated OVX rats (n = 8), ii) alendronate 3 mg/kg orally (n = 8), iii) La(XT) 100 mg/kg orally (n = 8).

Body weight and food consumption were monitored weekly. At the end of the study, the animals were euthanized by cardiac puncture under isoflurane anesthesia. One femur, one tibia and major organs including kidney, liver, spleen, lung, and brain were collected according to the protocol and stored at −80 °C until analysis.

#### 2.1.2 Experimental diets

All the treatment compounds were incorporated into the rat chow by Research Diet, Inc. La(XT) was incorporated at the highest dose level by Research Diet, Inc. Lower dose levels were achieved by mixing treated rat chow with basic rat chow in a V-blender in-house to achieve the desired dilution. Dosage calculations were based on an average food consumption of 30 g/day, and average weight of 0.3 kg/rat. The alendronate group received 0.03 g alendronate/kg basic research diet (Pytlik et al., 2004; Bolek and Korzeniowska, 2012). The La(XT) 100 mg/kg group received La(XT) 1 g/kg mixed into the basic diet.

#### 2.1.3 Biochemistry profile

Blood samples were collected into 4 mL BD vacutainers at baseline (1 week), middle (7 weeks) and at the end of the study (13 weeks). Plasma was separated by centrifugation at 1500 ×*g* for 10 min and stored at −80 °C until analysis. Plasma biomarkers, including calcium, phosphorus, creatinine, ALT and AST, were determined by Prairie Diagnostic Services Inc. (Saskatoon, SK, Canada).

#### 2.1.4 Histology

Kidney, liver, brain, lung, and spleen samples were placed immediately in 10% neutralized formalin at least 20 times the volume of the tissues. Histological slice and staining were prepared by Prairie Diagnostic Services Inc. A veterinary pathologist performed a blinded histological evaluation on each organ.

#### 2.1.5 Micro-CT imaging of tibia

Microstructural properties of the right tibia were determined using a Skyscan 1172 micro-CT system (Bruker, Massachusetts, USA), using an isotropic voxel size of 10 μm. Images were acquired using an X-ray source setting of 70 kVp/142 μA with a 0.5 mm aluminium filter. A 180 degree scanning was used with 4 frame averaging, 0.3° rotation steps and 100 ms exposure time. A total of 24 tibiae (8 bones from each group) were tested for imaging. Bones were rescanned if the image was not clear. The images were reconstructed using NRecon software 1.7 (Bruker, MA, USA) and analyzed using CTAn 1.13 (Bruker, MA, USA). The regions of interest (ROIs) were identified starting at a distance of 0.5 mm to the growth plate and extended for another 2 mm. ROIs were calculated using a semi-manual interpolation method. In brief, trabecular bone regions were manually drawn on every 30th image throughout the image dataset, and then interpolated in between. The following indexes of the trabecular bone were chosen to evaluate the bone microstructure: bone volume fraction (BV/TV, %), trabecular thickness (Tb.Th, μm), trabecular separation (Tb.Sp, μm) and trabecular number (Tb.N, 1/μm). BV/TV represents the volume of mineralized bone per unit volume of the bone sample. Tb.Th is the local thickness of cancellous structure. Tb.Sp is the spacing between two trabecular structures. Tb.N is the inverse of mean distance between two cancellous structures.

#### 2.1.6 Mechanical test of tibia

**Fig. 1:**
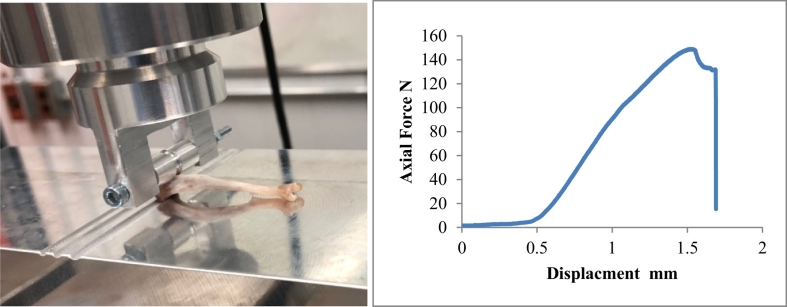
Metaphyseal three-point bending test setup (left) and load-displacement bending curve (right). The end of dorsal proximal tibia was placed in the appropriate notches. The loading position was determined by measuring a distance of exactly 3 mm between the end of proximal tibia and center of the roller stamp. The right figure showed the typical load-displacement bending curve. The loading to failure was conducted with a displacement rate of 10 mm/min. Testing ceased when fracture occurred or when 2 mm of cross-head displacement or 200 N of load had been reached.

We evaluated metaphyseal tibial failure load and stiffness using a previously validated 3-point bending test presented by McElroy and Wade (1987). The same tibiae (n = 8 for each group) were used for mechanical test. Due the nature of the test, each bone can only tested once. For this test, tibiae were positioned against a notched aluminium alloy block, with the end of the dorsal proximal tibia placed in the notches while the distal tibia was free to slide. Loading was applied using a servohydraulic material testing system (MTS Bionix, 250 N load cell). Load was applied via a roller stamp positioned 3 mm from the end of the proximal tibia (without the epiphysis). A 1 N preload was first applied, then loading to failure was conducted with a displacement rate of 10 mm/min. Testing ceased when fracture occurred or when 2 mm of cross-head displacement or 200 N of load had been reached. Load-displacement data were recorded and evaluated using custom algorithms (Matlab, Natick, MA). Failure load was defined as the maximum load prior to fracture. The slope of the most linear portion of the load-displacement curve prior to yielding was used to define stiffness (Fig. 1).

### 2.2 Statistical analysis

The experimental data were plotted and analyzed using one-way ANOVA with Tukey's post hoc test in GraphPad Prism (version 8.0, CA, US). Data were presented as mean with standard deviation. Significance was set at p ≤ 0.05.

## 3 Results and discussion

We previously conducted a 4-week study of La(XT) administration in healthy SD female rats to determine the toxicological profile and tissue distribution of La(XT) following oral and intravenous administration. We found La(XT) preferentially accumulated in the bone in a dose-dependent manner. No significant liver or kidney toxicities were observed in the previous study (Weekes et al., 2017). The purpose of the present study was to further evaluate the safety profile and the effect of La(XT) in OVX rat model of osteoporosis. We found that La(XT) was well tolerated in experimental animals following three months of treatment.

### 3.1 Basic characteristics of experimental animals

**Fig. 2:**
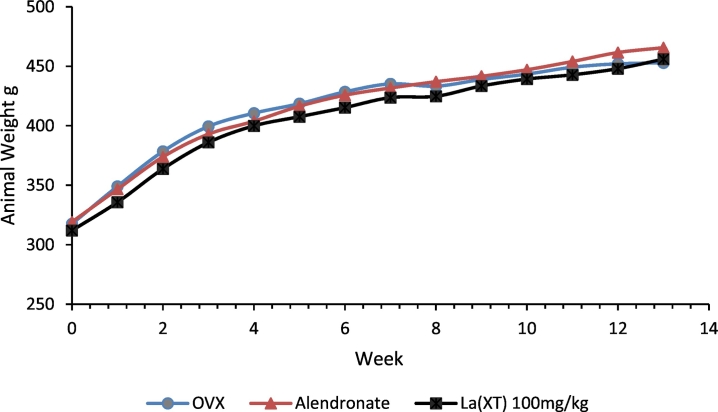
Animal weight growth chart. All experimental rats steadily gained weight through the 12-week study. No statistical difference in weight was observed between treatment groups (p > 0.05, n = 8).

As shown in Fig. 2, All OVX rats exhibited rapid weight gain after surgeries, then slowed down after 6 weeks, which was consistent with literature evidence that OVX rats gain weight following ovariectomy (McElroy and Wade, 1987). Mean weekly food consumption per cage was similar between animals fed with the basic diet and the diets containing the treatment compounds (Data not shown). No differences in food intake between experimental groups were observed through the entire study. Our data suggest dietary supplementation of lanthanum compounds did not affect food intake or animal weight gain.

### 3.2 Biochemistry profile

**Fig. 3:**
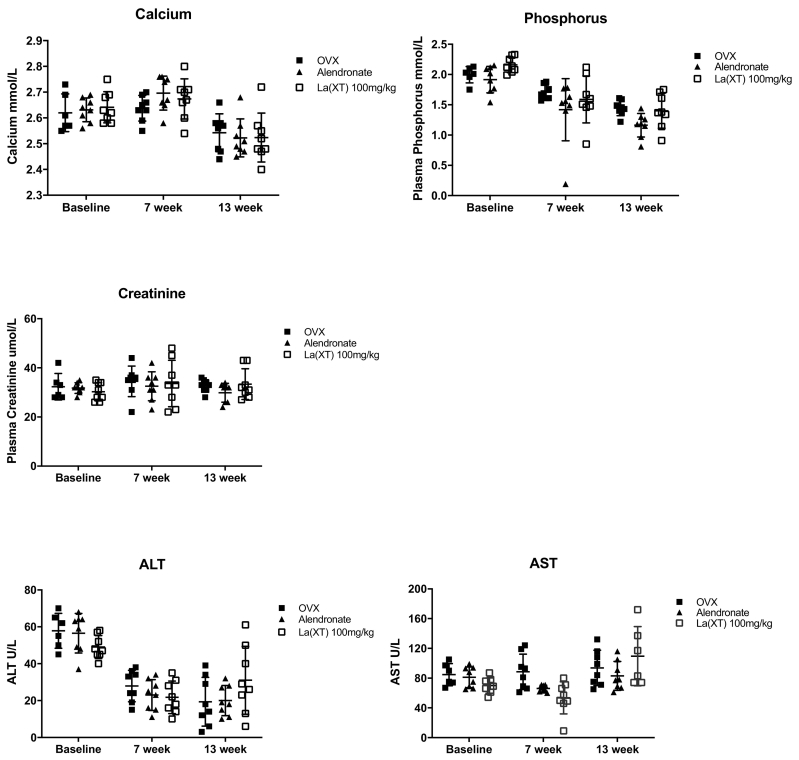
The effect of lanthanum on serum calcium, phosphorus, creatinine, AST and ALT in sham and OVX rats at baseline (week 1), middle (week 7) and end of study (week 13). Data were presented as mean ± STD. No significant differences were found between treatment groups at each time point (p > 0.05, n = 8). ALT: alanine aminotransferase, AST: aspartate aminotransferase.

Levels of plasma calcium, phosphorus, creatinine, ALT, and AST were determined. The animals enrolled in this study were supplied with standard rodent diet containing elemental calcium and phosphorus. The literature suggests that a decrease in plasma calcium level and an increase in phosphorus level in OVX rats with osteoporosis, we observed no change regarding plasma calcium and phosphorus levels in the current study (Fig. 3) (Elkomy and Elsaid, 2015; Aminah et al., 2017). It was not clear that why the phosphorus level declined in all experimental animals during the study. Alendronate treatment had a moderate effect on plasma levels of phosphorus (p = 0.0277) in OVX rats, although evidence showed that alendronate was able to effectively normalize plasma levels of calcium and phosphorus (Fahmy et al., 2015).

It is known that lanthanum is excreted predominantly by the liver (Toussaint et al., 2020). Our previous 4-week study of La(XT) administration in rats detected up to 136.1 μg/g La^3+^ in rat liver (Weekes et al., 2017). Taking into consideration that La^3+^ accumulates in the liver, AST and ALT levels were measured in the current study to assess potential hepatotoxicity. No significant differences in plasma levels of ALT and AST were observed over the study period between treatment groups, similar to our preliminary study (Weekes et al., 2017). Creatinine is used as a marker of renal glomerular function. During the study period, serum creatinine levels did not differ between groups. These results are consistent with a prior study in rats in which 22 weeks of treatment with a diet supplemented with 2% (w/w) La_2_(CO_3_)_3_ did not result in changes in serum creatinine (Damment et al., 2011).

Histological assessments were performed by a veterinary pathologist blinded to treatment. No remarkable changes were reported between treatment and control groups in kidney, liver, spleen, lung and brain. The histological findings in these organs were consistent with plasma biomarker results, in that there were no differences between treatment groups or between drug-treated and control groups. Our data suggest that the oral supplementation of La(XT)100 mg/kg showed no obvious toxicity in this study according to the measured parameters.

### 3.3 Micro-CT imaging

**Fig. 4:**
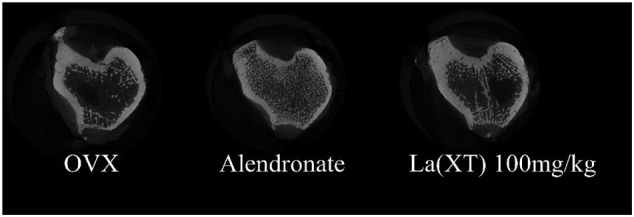
Representative cross-sectional micro-CT images of proximal tibia in sham and OVX rats. Images were acquired using X-ray source setting of 70 kV/142 μA with a 0.5 mm aluminium filter for noise reduction. A 180 degree scanning was chosen with an averaging frame of 4, 0.3° rotation steps and 100 ms exposure time. These pictures were selected at a distance of 1.5 mm from growth plate.

**Fig. 5:**
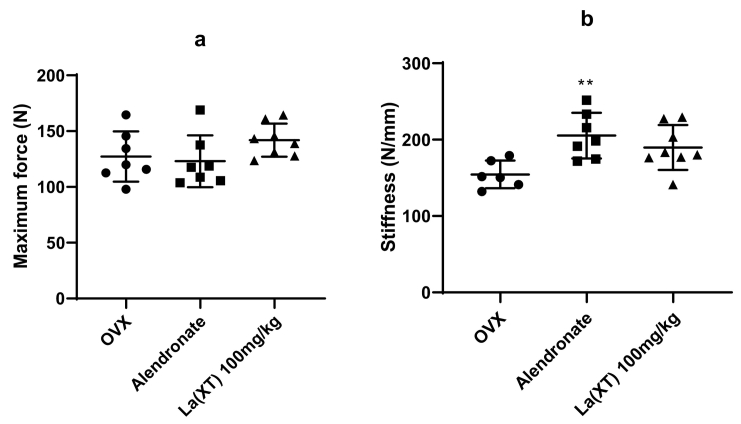
Comparison of maximum force (N, a) and stiffness (N/mm, b) in sham-operated (Sham) and ovariectomized (OVX) groups. Experimental data were analyzed using one-way ANOVA with Tukey's post hoc test. Asterisk indicates p < 0.05 for the values of the treatment groups compared with that of OVX only group (n = 6 or more).

Osteoporosis is characterized as loss of trabecular bone and deterioration of bone microstructure (Diab and Watts, 2013). Therefore, 3D histomorphometry was utilized using micro-CT to evaluate the potential effects of La(XT) treatment on trabecular bone microstructure following 13 weeks of dosing. Fig. 4 shows representative cross-sectional images from each group. BV/TV was significantly decreased in non-treated OVX rats, compared to the sham-operated control group after 3 months in the study, which indicated that the osteoporosis was successfully developed in the ovariectomized rats (Table 1). The mechanical analysis of the tibia further confirmed the development of osteoporosis (Fig. 5). Alendronate is a bisphosphonate used in treatment of osteoporosis, and has been demonstrated to enhance bone microstructure and strength in ovariectomized rats (Cho et al., 2017; Silva et al., 2020), and therefore used as a clinically relevant comparative baseline for the development of alternative strategies in the present study. In the current study, alendronate improved BV/TV by a factor of 1.28 (p < 0.0001), and decreased Tb.Sp by 71% (p = 0.0044), compared to the OVX only group.

**Table 1:** Bone parameters in the tibia metaphysis in sham (sham-operated) and OVX (ovariectomized) rats after three-month treatment using microCT imaging system. Statistics were calculated using ANOVA with Tukey's multiple comparison. Asterisk indicates p < 0.05 for the values of the treatment groups compared with that of OVX only group (n = 6 or more).

		OVX	Alendronate	La(XT) 100 mg/kg
BV/TV %	Mean	14.26	32.52	14.98
	STD	4.85	10.3	2.27
	p		<0.0001*	0.9745
	95% CI		−26.71 to −9.811	−9.175 to 7.723
Tb.Sp μm	Mean	518.57	147.18	488.37
	STD	200.69	34.84	94.66
	p		<0.0001*	0.8879
	95% CI		208.0 to 534.8	−133.2 to 193.6
Tb.Th μm	Mean	93.11	73.69	93.47
	STD	4.61	8.22	6.19
	p		<0.0001*	0.9808
	95% CI		11.22 to 27.62	−8.565 to 7.843
Tb.N 1/μm	Mean	0.00153	0.0043525	0.001594
	STD	0.00052	0.00096566	0.000155
	p		<0.0001*	0.9932
	95% CI		11.22 to 27.62	−8.565 to 7.843

Although it was not statistically significant, there was a trend in the BV/TV values in rats treated with increasing doses of La(XT). La(XT) 100 mg oral treatment increased BV/TV by 5.09% (p > 0.99). Similarly, Tb.Sp had decreasing trends of 5.82% (p > 0.99) in La(XT) 100 mg oral. It has been reported that intravenous injections of lanthanum chloride at doses of 5 mg/kg of La^3+^ for five weeks increased the trabecular bone mineral density (Miyagawa and Daimon, 2010); this represents a much higher dosage than the highest dose employed in our study, at 1 mg/kg IV (equivalent to 0.2185 mg La^3+^/kg), although we hypothesize that there is a continuous accumulation of La^3+^ in the bone over the 3 month study period.

La_2_(CO_3_)_3_ is an alternative source of La^3+^ to evaluate the effect of La^3+^ on bones in a studying using OVX Wistar Han rat model. It has been demonstrated that the BMD was significantly increased with 3-month dietary supplementation of 1.74 g/kg La_2_(CO_3_)_3_ (von Rosenberg and Wehr, 2012). As discussed previously, lanthanum exhibits poor solubility and undergoes extensive first-pass effect. The absorption of La^3+^ is extremely low in the form of La_2_(CO_3_)_3_ compared with La^3+^ in the form of La(XT). This is the important rational to design a more soluble lanthanum compound complex. More, it should be noted that in the OVX Wistar Han rat model, BMD was used to measure the areal density of bone, while, we used BV/TV to directly measuring the 3D structure of the trabecular bone. It is not practical to compare our results with the OVX Wistar Han rat model since different detection methods were chosen, thus we did not include La_2_(CO_3_)_3_ in the current study.

### 3.4 Mechanical tests

The sham group showed the highest maximum loading force value (170.6 ± 17.8 N) which was significantly higher than OVX group (127.2 ± 22.5 N) (p = 0.0013). Treatment of the rats with alendronate, or La(XT) did not show a higher maximum force compared to the OVX group. La(XT) treatment showed a trend toward higher maximum force, with highest values found in La(XT) 100 mg/kg (141.9 ± 14.9 N). Although they were not significant, these forces were 11.5% (p = 0.77) higher than the OVX group (Fig. 1).

In the analysis of stiffness, the mean value in the OVX group was 154 ± 18 N/mm. The alendronate and La^3+^ treatments improved stiffness compared to no treatment in the OVX rats. The stiffness in alendronate, and La(XT) 100 mg/kg groups were 205.3 ± 29.8 (p = 0.04), and 189.7 ± 29.3 (p = 0.3) N/mm. The Alendronate treatment group showed a 32% (p = 0.04) increase in stiffness than that of the OVX group. The mechanical properties of bone can be affected by many factors including loading speed, test location, bone size, and species (Oftadeh et al., 2015). It is therefore difficult to compare our findings with literature reports; nevertheless, our data suggest that administration of La^3+^ compounds preserved the mechanical properties of the tibia in OVX rats as measured by this test. We did not observe significant changes in loading force and stiffness of the tibia in OVX rats with increasing doses of La(XT); however, the trend could reach significance at a higher La(XT) dose or long duration of treatment in future study.

Based on our findings, 100 mg/kg/day of La(XT) was well-tolerated in OVX rats but may still fall below the pharmacological dose required to produce a significant effect against osteoporosis. The literature reports that in humans taking 375–3000 mg/day La_2_(CO_3_)_3_, the maximum concentration of La^3+^ in bone is 5.5 μg/g (Spasovski et al., 2006). This might be attributed to the extremely low bioavailability of La_2_(CO_3_)_3_ in humans. In an in vitro study it was shown that the La(XT) compound showed the greatest uptake into Caco-2 cell monolayers, which exhibit similar in vitro permeability characteristics as human intestinal tissue (Mawani et al., 2013; Meunier et al., 1995), which suggests at least some potential for permeability. However, only ~765 ng/g of La^3+^ was detected in the rat femora at a dose of 200 mg/kg for 4 weeks. This may be due to rapid systemic clearance, given that La(XT) has a half-life of only 4 h and falls below the detection limit after 6 h with a single IV dose of 1 mg/kg La(XT) (Cawthray et al., 2015). Nevertheless, we have previously demonstrated that La(XT) does accumulate in bone in a dose-dependent manner over time (Weekes et al., 2017). Our data suggest that to deposit sufficient amount of La(XT) in bone, we may need to modify the dosage regimen, or devise a formulation to improve oral bioavailability and thus bone accumulation.

Besides the limitations created by dosage choices and study duration, we have found that the OVX rat model used in this experiment may not be the most appropriate model to study human osteoporosis. Over the years, there have been multiple animal models being used in bone research according to their varied purposes. Ovariectomized rats are a popular selection among these choices due to the ease of mimicking the estrogen decline associated with postmenopausal osteoporosis, ease of administering drug compounds, and cost effectiveness (Komori, 2015; Lelovas et al., 2008). In contrast to human bones which undergo a dynamic bone remodeling cycle throughout the lifetime, rats do not undergo secondary cortical bone remodeling after reaching maturity (Clarke, 2008; Ross and Sumner, 2017). New perspectives indicate that the osteoporotic rabbit model derived by ovariectomy or glucocorticoid may be more suitable for studying human postmenopausal osteoporosis (Zhang et al., 2016; Wanderman et al., 2018). Rabbit bone has Haversian systems and reaches quick bone maturity at around 6 months (Permuy et al., 2019; Ravanetti et al., 2015). Similar to humans, rabbits possess active intracortical remodeling and a faster bone turnover which is favorable for studying compounds that affect bone remodeling balance (Mapara et al., 2012). Thus, utilizing a rabbit osteoporosis model in future studies may generate different results.

## 4 Conclusions

Our results demonstrated that La(XT) at the doses administered was well-tolerated in an OVX rat model of osteoporosis over a three-month chronic dosing study with no detection of kidney or liver toxicities. We observed a increasing trend of bone stiffness in the La(XT) 100 mg/kg group, compared to the control OVX group (p = 0.3). The absence of liver and kidney toxicity with drug treatment for 3 months, as well as the increased trabecular bone stiffness are encouraging for the pursuit of further studies with La(XT) for a longer duration of time.

## CRediT authorship contribution statement

All authors were involved in the design, execution and write-up of this manuscript.

## Declaration of competing interest

All authors listed in this paper declare no conflict of interest. Animal studies were approved by the University of Saskatchewan Animal Care Committee (Protocol #20150060), and performed in accordance with the guidelines outlined by the Canadian Council on Animal Care (CCAC). Funding for this study was provided by a 10.13039/501100000106Saskatchewan Health Research Foundation Establishment Grant (SHRF to K.M.W.). We acknowledge the Natural Sciences and Engineering Research Council (NSERC) of Canada for CGSM/CGSD fellowships (T.I.K.), the IsoSiM 10.13039/501100000038NSERC CREATE program at TRIUMF for their generous support (T.I.K.) and a Discovery Grant (C.O.).
